# Cannabinoids function in defense against chewing herbivores in *Cannabis sativa* L.

**DOI:** 10.1093/hr/uhad207

**Published:** 2023-10-13

**Authors:** George M Stack, Stephen I Snyder, Jacob A Toth, Michael A Quade, Jamie L Crawford, John K McKay, John Nicholas Jackowetz, Ping Wang, Glenn Philippe, Julie L Hansen, Virginia M Moore, Jocelyn K C Rose, Lawrence B Smart

**Affiliations:** Horticulture Section, School of Integrative Plant Science, Cornell University, Cornell AgriTech, Geneva, NY 14456, United States; Plant Biology Section, School of Integrative Plant Science, Cornell University, Ithaca, NY 14853, United States; Horticulture Section, School of Integrative Plant Science, Cornell University, Cornell AgriTech, Geneva, NY 14456, United States; Horticulture Section, School of Integrative Plant Science, Cornell University, Cornell AgriTech, Geneva, NY 14456, United States; Plant Breeding Section, School of Integrative Plant Science, Cornell University, Ithaca, NY 14853, United States; Department of Agricultural Biology, Colorado State University, Fort Collins, CO 80523, United States; Cirona Labs, Geneva, NY 14456, United States; Department of Entomology, Cornell University, Cornell AgriTech, Geneva, NY 14456, United States; Plant Biology Section, School of Integrative Plant Science, Cornell University, Ithaca, NY 14853, United States; Plant Breeding Section, School of Integrative Plant Science, Cornell University, Ithaca, NY 14853, United States; Plant Breeding Section, School of Integrative Plant Science, Cornell University, Ithaca, NY 14853, United States; Plant Biology Section, School of Integrative Plant Science, Cornell University, Ithaca, NY 14853, United States; Horticulture Section, School of Integrative Plant Science, Cornell University, Cornell AgriTech, Geneva, NY 14456, United States

## Abstract

In the decades since the first cannabinoids were identified by scientists, research has focused almost exclusively on the function and capacity of cannabinoids as medicines and intoxicants for humans and other vertebrates. Very little is known about the adaptive value of cannabinoid production, though several hypotheses have been proposed including protection from ultraviolet radiation, pathogens, and herbivores. To test the prediction that genotypes with greater concentrations of cannabinoids will have reduced herbivory, a segregating F_2_ population of *Cannabis sativa* was leveraged to conduct lab- and field-based bioassays investigating the function of cannabinoids in mediating interactions with chewing herbivores. In the field, foliar cannabinoid concentration was inversely correlated with chewing herbivore damage. On detached leaves, *Trichoplusia ni* larvae consumed less leaf area and grew less when feeding on leaves with greater concentrations of cannabinoids. Scanning electron and light microscopy were used to characterize variation in glandular trichome morphology. Cannabinoid-free genotypes had trichomes that appeared collapsed. To isolate cannabinoids from confounding factors, artificial insect diet was amended with cannabinoids in a range of physiologically relevant concentrations. Larvae grew less and had lower rates of survival as cannabinoid concentration increased. These results support the hypothesis that cannabinoids function in defense against chewing herbivores.

## Introduction

Secondary metabolism generates prolific variation in organic compounds across all taxa of life. In predominately sessile organisms, like terrestrial plants, secondary metabolites often function in mediating interactions with biotic and abiotic factors in the local environment [1, 2]. This biochemical diversity has expanded over evolutionary time as populations adapted to local ecosystems and species coevolved with new suites of interactors [3]. Cannabinoids, produced naturally in the greatest concentrations by *Cannabis sativa* L., are one such class of secondary metabolites that are derived from the enzymatically mediated convergence of the polyketide and plastidial isoprenoid pathways [4]. Biosynthesis and subsequent storage of cannabinoids in *C. sativa* are concentrated in glandular trichomes, hair-like structures that are most densely produced on pistillate inflorescences [5–7]. In the decades since cannabinoids were first isolated and characterized [8], more than 100 phytocannabinoids have been identified [9, 10]. Despite explicit research on these compounds being limited to the last century, there is evidence that *C. sativa* has been synthesizing cannabinoids for millions of years [11, 12] and that humans have used cannabinoids as medicine for several thousand years [13].

As an evolutionary strategy, cannabinoid biosynthesis almost certainly increased plant fitness. If this were not the case, natural selection would likely have purged, rather than maintained, the complex and metabolically costly pathway over millions of generations. Further, the independent evolution of cannabinoid biosynthesis in several plant lineages suggests important ecological functionality [14, 15]. The adaptive value of cannabinoid production is not definitively known, but prevalent theories include protection from herbivores, pathogens, or ultraviolet (UV) radiation [16, 17]. *C. sativa* is thought to have evolved in high-altitude environments, which has led many to postulate the function of cannabinoids as a photoprotectant against intense UV radiation [18–20]. However, recent studies have shown no difference, or a decrease, in cannabinoid concentration with supplemental UV radiation [21, 22], which has weakened support for this hypothesis. In support of the potential for cannabinoids to serve in defense against plant pathogens, antimicrobial properties have been demonstrated for several cannabinoids [23–25]. Despite this, research concerning the capacity of cannabinoids to suppress plant pathogens is limited [26, 27].

There is evidence that cannabinoids could provide defense against herbivores. McPartland [20] posits that cannabinoid production could have been an adaptation in response to the expansion of vertebrate herbivores like ungulates, rodents, and birds into the Eurasian steppe. There is also a clear mechanism of action for cannabinoids to deter such herbivores: in mammals and other species, tetrahydrocannabinol (THC) and other cannabinoids bind to cannabinoid receptors CB_1_ and CB_2_, members of a larger superfamily of G-protein-coupled receptors (GPCRs) [28]. These interactions and subsequent impacts on the nervous system could deter those herbivores from further feeding, resulting in reduced herbivore damage or preference.

Beyond vertebrates, there is evidence that cannabinoids mediate interactions with many taxa of insect herbivores, despite their lack of canonical cannabinoid receptors [29]. Rothschild and Fairbairn [30] demonstrated that the butterfly *Pieris brassicae* can distinguish between leaves sprayed with THC and cannabidiol (CBD) and that cannabinoid treatment affected moth oviposition behavior. Mantzoukas et al. [31] found that CBD had larvicidal action against *Tribolium confusum*, *Oryzaephilus surinamensis*, and *Plodia interpunctella*. Similarly, Park et al. [32] found that *Manduca sexta* larvae preferred to feed on leaves with lower levels of CBD, and increasing concentrations of CBD in artificial diet reduced larval size, weight, and survival. In contrast, He et al. [33] found that fruit flies (*Drosophila melanogaster*) developed a preference for food with various added phytocannabinoids, and Waser [34] observed only minor changes in ant (*Formica pratensis*) colony behavior when their diet was amended with THC. Beyond these, numerous studies have used *C. sativa* extracts as pesticides [35], though it is unclear what active compound or compounds mediated the response.

There is substantial biochemical variation in cannabinoids among populations and individuals of *C. sativa*. Historically, the *C. sativa* cannabinoid chemotype has been qualitatively classified based on the cannabinoid profile correlated with the status of two epistatic Mendelian loci: *B* and *O* [36–40]. Briefly, the *B* locus controls whether the dominant cannabinoid is THC, CBD, a combination of the two, or cannabigerol (CBG). The *O* locus functions biochemically upstream of the *B* locus and controls the capacity of the plant to produce cannabinoids in appreciable concentrations.

To establish whether cannabinoids play a role in plant defense against chewing herbivores, the segregating ‘Carmagnola’ × ‘USO-31’ F_2_ population [41] was leveraged as a common genetic background to conduct a series of lab- and field-based bioassays. The primary objectives of this study were to (1) determine whether plants with different concentrations of foliar cannabinoids incur corresponding levels of herbivore damage in the field, (2) quantify variation in larval feeding and growth on detached cannabinoid-competent versus cannabinoid-free leaves, and (3) determine if the addition of cannabinoids to artificial insect diet quantitatively affects larval survival and growth.

## Results

### Molecular markers, cannabinoid concentrations, and trichome morphology segregate individuals according to cannabinoid chemotype

The ‘Carmagnola’ × ‘USO-31’ F_2_ population [41] segregates in a similar fashion to populations described by de Meijer et al. [38]. Woods et al. [41], who first characterized the population, identified two major-effect epistatic loci that control cannabinoid concentrations: LG6.35 and LG9.40, which map to chromosomes 7 and 8 in the CBDRx reference genome [42], respectively. The LG6.35 locus corresponds to the previously mapped *B* locus [43], which can contain functional copies of tetrahydrocannabinolic acid synthase (THCAS) or cannabidiolic acid synthase (CBDAS) [42, 44]. Plants that are homozygous for the *B*_D_ haplotype, having a functional CBDAS and no functional THCAS, produce predominately cannabidiolic acid (CBDA) and are classified as chemotype III. When there is a homozygous knockout or knock-down of the functional cannabinoid oxidocyclase enzymes (CBDAS or THCAS) at the *B* locus, referred to as B_0_, plants accumulate cannabigerolic acid (CBGA) [37, 45, 46], the metabolic precursor of CBDA and THCA. This CBG-dominant phenotype is known as chemotype IV. The phenotypes of plants segregating at the LG9.40 locus correspond well to the phenotypes of the *O* locus described by de Meijer et al. [38] where *O*/*O* genotypes are fully cannabinoid-competent, *O*/*o* genotypes have significantly reduced cannabinoid concentrations, and *o*/*o* genotypes are essentially cannabinoid-free (<0.05% total cannabinoids). Cannabinoid-free individuals are classified as chemotype V.

**Figure 1:**
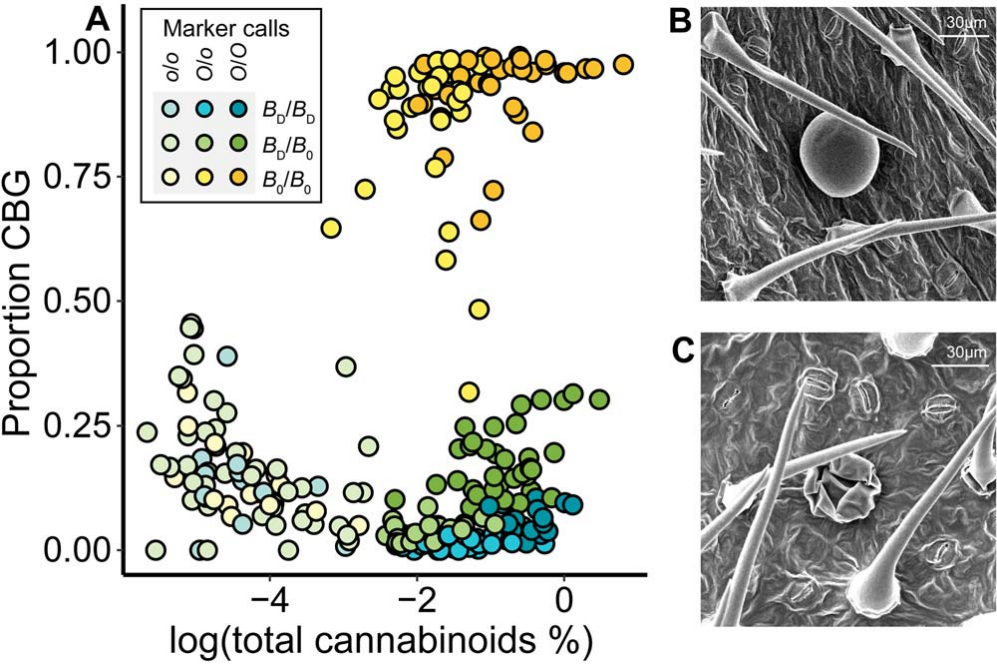
Variation in cannabinoid concentrations and trichome morphology in a segregating F_2_ population of *C. sativa*. (A) Visualization of foliar cannabinoid samples into three chemotypes when log(total cannabinoid %) is plotted against proportion CBG for 241 samples from plants grown in the field in NY. Colors indicate marker calls for the CH-OLS-V-1 and CH-USO31-IV-1 PACE assays. (B) Representative SEM image of a sessile glandular trichome from the abaxial surface of a cannabinoid-competent plant. (C) Representative SEM image of a collapsed sessile glandular trichome from the abaxial surface of a cannabinoid-free plant. In (B) and (C), bars indicate 30 μm.

To resolve the genotypes of the individuals in the F_2_ population, we developed PCR allelic competitive extension (PACE) genotyping assays to assess the allelic status of the *B* and *O* loci. Consistent with the previously described two-locus model [38, 40], the CH-OLS-V-1 and CH-USO31-IV-1 PACE assay results strongly correlated with the cannabinoid compositional data that separated the genotypes into three groups: CBD-dominant, CBG-dominant, and cannabinoid-free (Fig. 1A). To develop the CH-USO31-IV-1 assay, we amplified the CBDAS sequence from a CBG-dominant F_2_ individual and determined that it was 100% identical to a previously described CBDAS coding sequence (GenBank accession KP970860.1) [45].

To investigate potential correlations between trichome density and morphology with measured cannabinoid profiles or herbivory, scanning electron microscopy (SEM) was used to characterize the variation in the density of different trichome types among cannabinoid chemotypes. There were no significant differences in sessile glandular trichome density by cannabinoid chemotype (*F*(2,52) = 1.05, *P* > .05) (Fig. S1A). Notably, there were more sessile glandular trichomes on the abaxial surface of the leaf than the adaxial surface of the leaf (*F*(1,52) = 48.89, *P* < .001) (Fig. S1A). There was a difference in the morphology of the sessile glandular trichomes by cannabinoid chemotype, such that the trichomes appeared to be shrunken and collapsed in the cannabinoid-free individuals relative to the CBD- and CBG-dominant individuals (Fig. 1B and C). This morphology was also observed using light microscopy confirming that it was not an artifact of sample preparation for SEM.

### Cannabinoid-free genotypes sustained more herbivore damage than cannabinoid-competent genotypes in the field

**Figure 2:**
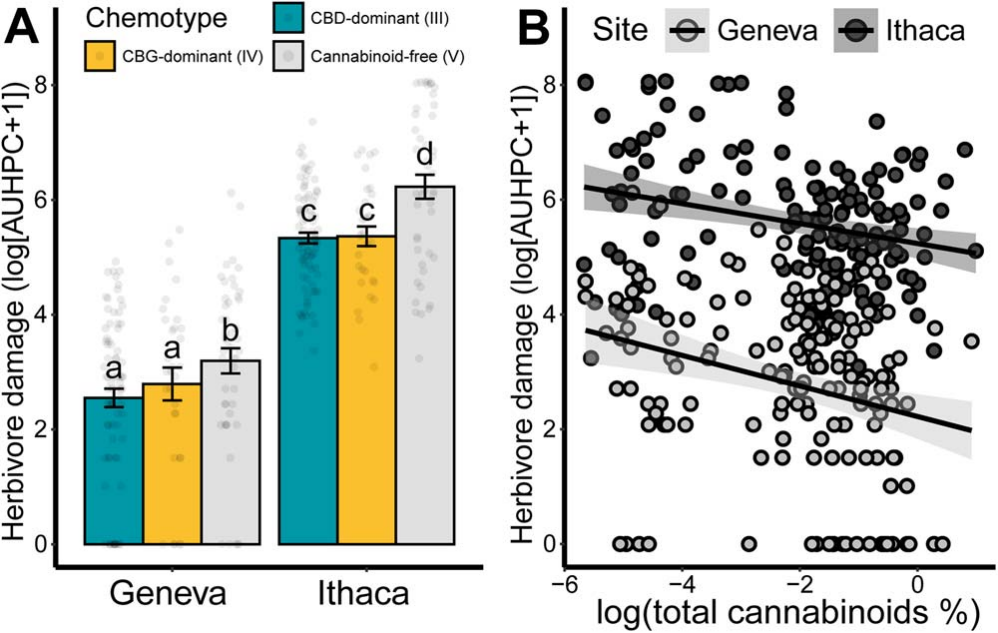
Herbivore damage on an F_2_ population of *C. sativa* segregating for cannabinoid chemotype. (A) Herbivore damage, log of area under the herbivory progress curve (AUHPC), by cannabinoid chemotype and field site. Herbivore damage was modeled using a mixed-effects model with site and cannabinoid chemotype as main effects and propagation method as a random effect. Letters indicate pairwise differences between chemotype-site groups based on a post-hoc multiple comparison using emmeans. Bars indicate means and error bars indicate standard error. (B) Correlation between total foliar cannabinoid concentration and herbivore damage by site. Regression lines are for a mixed-effects linear model with log(total cannabinoids %) and site as main effects. Light-shaded dots indicate plants in the Geneva trial, while dark-shaded dots represent plants in the Ithaca trial.

To determine if plants with different concentrations of foliar cannabinoids sustain different levels of herbivore damage, the segregating ‘Carmagnola’ × ‘USO-31’ F_2_ population was planted at two field sites and herbivore damage was rated weekly over the course of 5 weeks after transplant. There was a significant effect of site (*F*(1,312.02) = 377.45, *P* < .001) and cannabinoid chemotype (*F*(2,312.15) = 10.75, *P* < .001) on herbivore damage in the field (Fig. 2A). The testing for pairwise differences between chemotype-site combinations showed that at each site the cannabinoid-free group incurred significantly more herbivore damage than both the CBD-dominant and CBG-dominant groups (Fig. 2A). There was not a significant difference in herbivore damage between the CBD-dominant and CBG-dominant groups at either site (Fig. 2A). The log of total cannabinoid concentration was also correlated with herbivore damage at both sites (*F*(1, 262.31) = 7.95, *P* = .005) (Fig. 2B). While there was a main effect of site (*F*(1, 292.61) = 342.80, *P* < .001), there was not a significant interaction between site and the log of total cannabinoid concentration (*F*(1, 291.13) = 3.21, *P* > .05) (Fig. 2B).

During field ratings, the following insects were observed feeding on the plants: 24 instances of Japanese beetles (*Popillia japonica*), 3 of western black flea beetles (*Phyllotreta pusilla*), 2 of potato leaf hopper (*Empoasca fabae*), and 1 of tarnished plant bug (*Lygus lineolaris*). There was also damage on some plants that was consistent with slug feeding; however, their presence was not confirmed.

### Larvae feeding on cannabinoid-free genotypes performed better than those feeding on CBD-dominant genotypes

**Figure 3:**
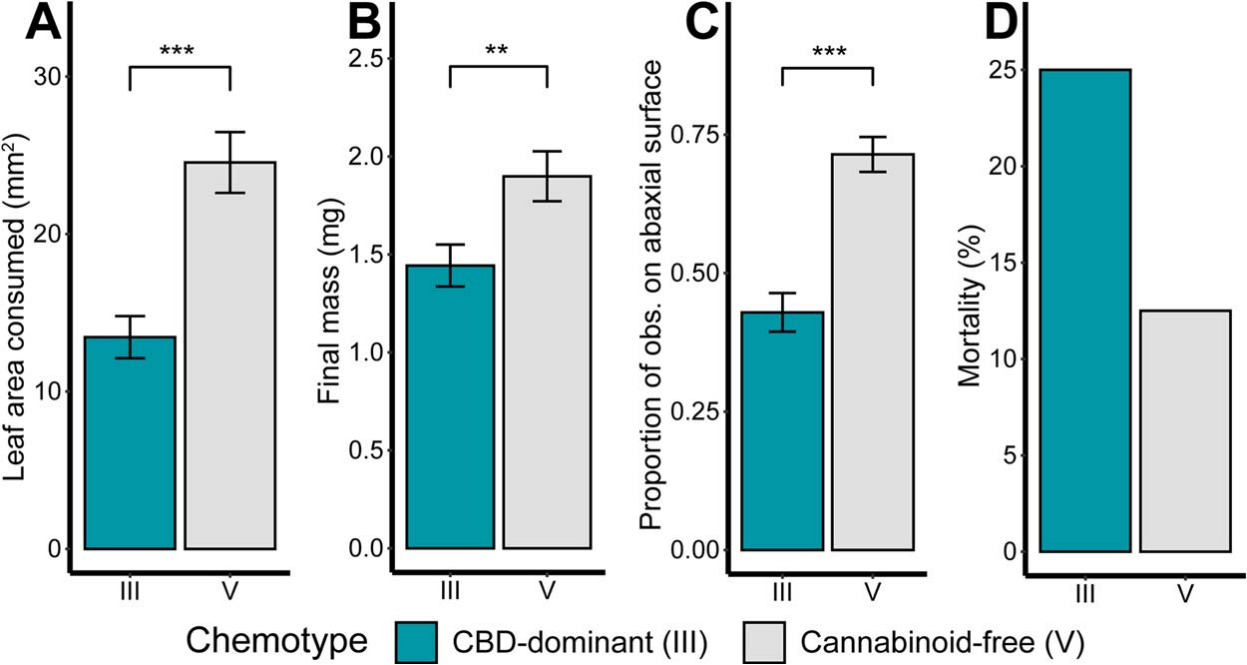
Results from detached-leaf bioassay of *T. ni* feeding on hemp leaves for 6 days. Leaves were collected from 12 genotypes: 6 cannabinoid chemotype III (CBD-dominant) and 6 chemotype V (cannabinoid-free). (A) Leaf area consumed after 6 days. (B) Final larval mass after 6 days. (C) Proportion of daily larval observations on the abaxial leaf surface. (D) Cumulative mortality (%) after 6 days. Colors indicate the leaf cannabinoid chemotype. Bars indicate means and error bars indicate standard error. Asterisks indicate significant differences between treatment groups based on a mixed-effect model with cannabinoid chemotype as a fixed effect and rep as a random effect (^**^, *P* < .01; ^***^, *P* < .001).

To quantify feeding and growth of herbivores on leaves of different cannabinoid chemotypes, a detached leaf bioassay was conducted using cabbage looper (*Trichoplusia ni*) larvae feeding on 12 unique cutting-propagated F_2_ genotypes: 6 CBD-dominant and 6 cannabinoid-free. Larvae consumed more leaf area of the cannabinoid-free genotypes than of the CBD-dominant genotypes (*F*(1, 113.05) = 25.33, *P* > .001) (Fig. 3A). Additionally, the larvae had a greater final mass feeding on the cannabinoid-free genotypes than feeding on the CBD-dominant genotypes (*F*(1, 113.07) = 8.72, *P* = .004) (Fig. 3B). Larvae feeding on cannabinoid-free genotypes were also more likely to be observed on the abaxial surface of the leaf surface than those feeding on CBD-dominant genotypes (*z* = 5.00, *P* < .001) (Fig. 3C). Twice as many of the larvae died feeding on the CBD-dominant genotypes than the cannabinoid-free genotypes; however, the cannabinoid chemotype only approached significance as a predictor of larval survival (*z* = −1.93, *P* = .054) (Fig. 3D).

### Increasing concentrations of CBDA and CBGA in artificial diet decreased larval growth and survival

**Figure 4:**
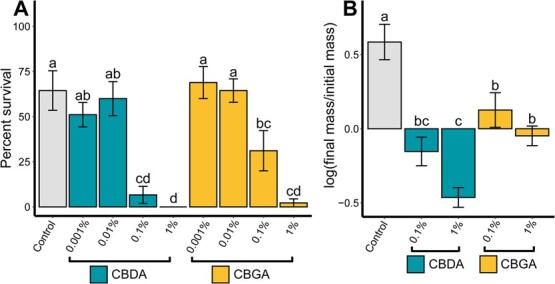
Results from artificial diet bioassay of *T. ni* feeding on diet amended with different concentrations of cannabinoids. (A) Variation in percent of neonate *T. ni* larvae surviving after 7 days feeding on artificial diet with different concentrations of cannabinoid emulsions painted on the surface. (B) Variation in growth of 7-day-old *T. ni* larvae surviving after 7 days feeding on artificial diet with the indicated concentrations of cannabinoid emulsions incorporated into the diet. Control in both experiments was artificial diet with added glycerin, the carrier for the cannabinoid emulsions. Bars indicate means and error bars indicate standard error of the mean. Letters indicate significant pairwise differences between groups within panels as determined by a Tukey’s HSD test.

To distinguish the effect of cannabinoids independent from other confounding factors, *T. ni* larvae were reared on artificial diet with various concentrations of cannabinoids painted on the surface of the diet or integrated into the diet. In the bioassay where cannabinoid emulsions were painted on the surface of the diet, there was a significant effect of treatment on larval survival after 3 days (*F*(8,63) = 18.56, *P* < .001) (Fig. 4A). Emulsion concentrations greater than 0.1% of CBDA or CBGA resulted in significantly lower larval survival than the control. None of the 45 larvae feeding on the 1% CBDA treatment were alive after 3 days. In the bioassay where cannabinoid emulsions were incorporated into the diet, there was a significant effect of treatment on the log of the final to initial mass ratio after 7 days (*F*(4,110) = 16.71, *P* < .001) (Fig. 4B). All the treatment groups had a significantly lower log of the final-to-initial mass ratio than the control group.

## Discussion

The palliative and intoxicating effects of cannabinoids in humans have inspired the sustained cultivation of *C. sativa* for thousands of years and intentional selection for greater concentrations of cannabinoids. For millions of years before that, the primary benefit of cannabinoid production to the plant is thought to have been as a defense mechanism. The results reported here demonstrate that CBDA and CBGA, two of the most abundant phytocannabinoids produced by *C. sativa*, can reduce growth and survival of chewing herbivores independent of other biochemical or physical factors.

### Cannabinoids reduce foliar herbivore damage in the field

Cannabinoid production is beneficial to plants in a field setting through reduction in chewing herbivore damage. It was evident by visual inspection that herbivores strongly preferred feeding on some plants in the population more than others. Several of the plants were lethally defoliated within the first 2 weeks after transplant, all of which were cannabinoid-free, while adjacent plants had little damage. Most of the damage is thought to be a result of *P. japonica* feeding, as it had the greatest number of sightings and the damage observed on most plants was consistent with damage on leaves actively being consumed by *P. japonica*. As large, active, and mobile herbivores, it is reasonable to conclude that the differences in herbivore damage were at least in part a result of herbivore preference for cannabinoid-free genotypes. While we cannot rule out that other secondary compounds or physical deterrents may play a role, the large number of segregating F_2_ individuals evaluated would likely reflect a randomization of those other traits in the shared genetic background unless they were segregating together with the cannabinoid chemotype.

While the plants that died from defoliation had conclusively lower fitness than those that survived, the degree to which the significant loss in photosynthetic area led to reduction in plant fitness among surviving cannabinoid-free plants was not characterized. Maintenance and fixation of the cannabinoid-free phenotype in hemp cultivars was the result of breeding and intentional selection in a Ukrainian fiber hemp breeding program for plants that produced very low concentrations of cannabinoids [38]. Without artificial selection, it seems unlikely that cannabinoid-free plants would compete and survive to comprise substantial proportions of wild or cultivated populations. Population genetics studies of existing wild and feral populations of *C. sativa* would provide additional information about the maintenance of cannabinoid-free individuals in proportion with individuals of cannabinoid-competent chemotypes in populations under natural selection.

### Cannabinoids alter larval consumption, growth, and behavior on detached leaves

Several studies have previously investigated the impact of cannabinoids on insect preference, performance, and behavior [30–34, 47, 48]. Even though it was not observed as part of the herbivore community in the field experiments, *T. ni* was selected as a model due to its wide use as a model herbivore, its broad host range as a generalist, including over 160 plant species from 36 families [49], and its historic distribution throughout Eurasia, where *C. sativa* is thought to have originated [50, 51].

By conducting detached leaf bioassays with single *T. ni* larvae, performance on leaves from cannabinoid-free and CBD-dominant genotypes could be directly contrasted while limiting confounding environmental factors. Consistent with the trend observed in the field experiment, the larvae consumed less leaf area of CBD-dominant leaves than of cannabinoid-free leaves. This indicates that the mechanism by which cannabinoids deter herbivory is more than just non-preference as identified in the choice bioassay conducted by Park et al. [32]. The presence of, contact with, or consumption of cannabinoids reduces leaf consumption even if there are no other sources of food available. Additionally, assuming that the variation in cannabinoid concentration was greater than any variation in other nutrients, these results are consistent with Bolt et al. [52] who observed a negative correlation between cannabinoid:N ratios and herbivore performance.

### Increasing concentrations of CBDA or CBGA in artificial diet proportionally reduced larval performance

The addition of CBDA and CBGA in concentrations greater than 0.1% to artificial diet through surface painting or integration quantitatively reduced larval performance. This result is consistent with the results of previous studies adding CBD to *M. sexta* [32] and fall armyworm (*Spodoptera frugiperda*) [48] artificial diet. While significant differences were observed in both experiments, the larvae were only reared on this diet for 6–7 days so the potential impacts of cannabinoids on molting, pupation, and adulthood are not known. There were no significant differences in survival between CBDA and CBGA treatment groups of the same concentration for the surface painting bioassay. However, the 1% CBDA treatment group in the integration bioassay had a significantly lower log of the final-to-initial mass ratio than the 1% CBGA.

In addition to CBDA and CBGA, *C. sativa* produces numerous structurally diverse cannabinoids [9, 10]*.* The phenomenon of prolific biochemical diversification is not unique to cannabinoids, being observed in numerous classes of secondary metabolites and lineages of plants [2]. The screening hypothesis proposes that proliferation and maintenance of biochemical diversity is advantageous because, given that most compounds will not be biologically active against a given organism, producing many compounds confers a reasonable probability that some will be active against the suite of organisms a plant encounters [53]. This hypothesis predicts a species-specific response to different cannabinoid compounds, which has been observed in several insect species [30, 33].

An important question is whether the activity of acid-form cannabinoids differs from their non-enzymatically produced neutral counterparts, as is the case for mammalian cannabinoid receptors [54]. In living plants, the vast majority of the cannabinoid pool is present in the acid form, but most studies to date have tested the effects of decarboxylated cannabinoids. This study clearly demonstrates that the acidic cannabinoids produced *in planta* have insecticidal activity when used in feeding studies. A more comprehensive study of different cannabinoid compounds is needed to determine whether some are more potent herbivore deterrents than others and whether there is variable activity of different compounds among herbivore species.

### Cannabinoid-free plants have collapsed sessile glandular trichomes

Synthesis and storage of structurally diverse classes of secondary metabolites in glandular trichomes is not unique to *C. sativa*. Being elevated from the surface of the plant, trichomes are in an optimal position to make first contact herbivores and pathogens while also maintaining physical separation of potentially toxic compounds from the leaf surface [55]. Many classes of bioactive secondary metabolites are produced in glandular trichomes including terpenoids [56], phenylpropanoids [57], flavonoids [58], and acyl sugars [59].

As the predominate, if not exclusive, site of cannabinoid biosynthesis in *C. sativa*, glandular trichome morphology has been a focus of research for the last half century [7, 60, 61]. In both *C. sativa* [38] and tomato (*Solanum lycopersicum*) [62], variation in trichome morphology has been associated with variation in trichome-produced biochemicals. Variation in trichome morphology could also be a key factor in function as a mechanical defense, physically restricting herbivore movement and feeding [63].

Consistent with the headless phenotype of stalked glandular trichomes in cannabinoid-free inflorescences described by de Meijer et al. [38], leaves of cannabinoid-free plants were observed to have deformed sessile glandular trichomes. The collapsed appearance of the trichomes on cannabinoid-free genotypes suggests that they had been filled prior to collapse; otherwise, the cuticle would not have fully expanded. This could have occurred through the accumulation of terpenoids or other compounds that could be subsequently translocated, metabolized, or volatilized from the trichomes. The consistency of sessile glandular trichome density among cannabinoid chemotypes indicates that the phenotype is not related to the regulation of trichome production, initiation, or development, but rather the trichome contents. More research is needed to determine the mechanism of trichome collapse and how this phenotype is related to the very low concentration of cannabinoids in these plants.

Because the collapsed trichome phenotype was confounded with the cannabinoid-free trait *in planta*, it was impossible to resolve whether the difference in feeding in the field and on detached leaves was a result of the lack of cannabinoids, the reduced size of physical impediments on the leaves, or another unmeasured factor correlated with the two. The isolation of cannabinoids in the artificial diet assays provides evidence that cannabinoids can alter herbivore growth and survival independent of other factors, but this does not resolve whether the variation in trichome morphology could be an additional mediator of plant–herbivore interactions on intact leaves. The avoidance of the abaxial leaf surface on CBD-dominant genotypes, which has a greater density of sessile glandular and cystolithic trichomes, by larvae in the detached leaf bioassay could indicate an effect of trichome morphology or cannabinoid abundance on larval behavior.

### Potential mechanisms for cannabinoid–insect interaction

The apparent evolutionary loss of canonical cannabinoid receptors in insects [29], which have been conserved in species as diverse as mammals, birds, amphibians, fish, mussels, and *Hydra*, is puzzling. Logically, this leads to the question how do cannabinoids impact insect preference and performance if not through these receptors? Although more research is necessary, the CB_1_/CB_2_-independent action of cannabinoids in insects could be through affinity for other conserved GPCRs known to interact with cannabinoids, such as GPR55, TPRV channels, or PPAR-γ [54]. Further, Abendroth [48] found that the rearing *S. frigipeda* on artificial diet with increasing concentrations of CBD resulted in decreased protease and cytochrome P450 activity and increased β-glucosidase activity, which could provide further insight into intermediate steps in the cascade between a cannabinoid–receptor interaction and changes in insect preference and performance. Other potential mechanism have been described by Koch [47] who found that CBD disrupts exoskeleton formation in *M. sexta* and can result in lethal molting failure and Park et al. [32] who found CBD altered neural activity of *M. sexta*. The mechanistic studies to date have used lepidopteran larvae as a study system, but more research is needed to determine if the mechanism of action is conserved across all lepidopterans and if other mechanisms exist in diverse clades of arthropods with different modes of feeding.

### Future directions

We are far from having a complete understanding of the function of cannabinoids in plant defense. Despite growing evidence of cannabinoids functioning as defenses against herbivores, there are numerous herbivores that feed on *C. sativa* [64–67]. In other plant–herbivore systems with specialized classes of secondary metabolites, specialist insects have coevolved to tolerate, avoid, or even co-opt these defenses [68]. Curiously, many of the herbivores that feed on *C. sativa* are generalists and some, like corn earworm (*Helicoverpa zea*), feed directly on cannabinoid-rich inflorescences [69]. Based on the results of this study, one prediction is that polyphagous herbivores feeding on cannabinoid-competent *C. sativa* would perform better on cannabinoid-free *C. sativa*. Additionally, it is unclear if and how cannabinoids could affect piercing-sucking herbivores like aphids and mites that, unlike chewing herbivores, do not directly ingest glandular trichomes. One limitation of this study was that both field sites were located in New York and that the field ratings only covered a portion of the growing season. Field studies in other locations and at other points during the growing season would provide insight into the impact of cannabinoids on different herbivore communities.

Another consideration for investigating the function of cannabinoids is tissue-specific variation in concentration, specifically in inflorescences. It is logical that cannabinoids would be concentrated in tissues proximal to those producing seed and thus may be under strong selective pressure through influencing seed survival, dispersal, and subsequent reproductive success contributing to plant fitness [70]. Further, when considering the function of cannabinoids in defense against herbivores, the difference in concentration between staminate and pistillate inflorescences in *C. sativa* may provide insight into an evolutionary driver of dioecy, for which ecological factors like herbivory and pollination are thought to play a role [71, 72].

The function of cannabinoids as a defense against herbivores is not mutually exclusive with the other hypothesized functions. Broadening our understanding of the scope and mechanism(s) of action of this emerging class of plant defenses will deepen the existing body of knowledge encompassing ecologically functional plant metabolites.

## Materials and methods

### Field-based herbivory bioassay

To test if *C. sativa* plants with different cannabinoid profiles would sustain different levels of chewing herbivory, we first planted progeny from a segregating F_2_ population of CBD-dominant, CBG-dominant, and cannabinoid-free plants at two field sites in New York State. For the field experiments, plants were either propagated from cuttings of greenhouse-grown clonally maintained genotypes or were germinated directly from seeds and transplanted into the field. Cutting propagated genotypes were replicated twice at each of the two sites.

All of the genotypes evaluated were part of a segregating F_2_ population derived from a cross between ‘Carmagnola’ and ‘USO-31’ [41]. Initially, 100 individuals were planted in a greenhouse in Geneva, NY in September of 2020. Following establishment, leaves were sampled from 96 individuals and cannabinoids were quantified using high-performance liquid chromatography (HPLC) following the methods described by Stack et al. [40]. Based on these data, plants of chemotypes III, IV, and V were selected to be maintained clonally in the greenhouse under 18:6 (L:D) with a 1-hour night break to prevent the plants from flowering. On 10 June 2021, two-node cuttings were rooted from the greenhouse stock plants in OASIS® Rootcube® wedges (OASIS® Grower Solutions, Kent, OH) using Clonex® rooting gel (Hydrodynamics International, Lansing, MI, USA).

Four hundred seeds from the same F_2_ population were planted in the first week of June, 2021. Two hundred and eighty individuals germinated and were randomly assigned to the two field sites such that 140 seedling-propagated individuals were transplanted at each site. Seedlings and two cuttings of each clonally propagated genotype were randomized and transplanted into raised beds covered with black plastic mulch with drip irrigation at each site during the last week of June. Landscape fabric was used to control weeds in the alleys.

Percent herbivory was rated visually by a single person once per week on a scale of 0%–100% total leaf area consumed for 6 weeks. For each plant, the rater estimated the total number of leaves and the proportion of leaf area consumed for leaves with herbivore damage before determining a plant-level rating. For example, if a plant had ~50 leaves and 10 of them had ~50% leaf area consumed, that would be a rating of 10%. Ratings did not always increase week to week as many plants produced new leaves more quickly than the leaves were being consumed. If a plant died as a result of complete defoliation, the rating was maintained at 100% for subsequent weeks. Images of cannabinoid-free and CBD-dominant plants 2 weeks after planting can be found in Fig. S2. The area under the herbivory progress curve (AUHPC) was calculated with the ‘audpc’ function from the R package agricolae [73] to integrate the intensity across the 5 weeks. Herbivores on the plants were noted whenever they were observed.

On 21 July, ~1 month after transplant, the two most recently fully expanded leaves (approximately three nodes from the apical meristem) were sampled to quantify foliar cannabinoid concentration. Cannabinoids were quantified in the leaf samples following the protocol above with the following modifications: 200 mg of homogenized tissue was used to increase the precision of the protocol to detect very low concentrations of cannabinoids. For cutting-propagated genotypes that had died as a result of defoliation or otherwise could not be sampled, total cannabinoid concentrations were imputed based on foliar cannabinoid concentrations from other individuals of the same genotype.

### SEM and light microscopy

Fully expanded hemp leaves were harvested from plants maintained in the greenhouse under the above conditions. Two representative plants of the CBD-dominant, CBG-dominant, and cannabinoid-free chemotypes were selected and five leaves were collected from each plant. Abaxial and adaxial leaf sections measuring 1 cm × 1 cm were prepared for SEM by adhering them to 12.7-mm pin stubs (EMS75710, Electron Microscopy Sciences, Hatfield, PA, USA) with adhesive tabs (EMS76760, Electron Microscopy Sciences). To preserve the sections, the pin stubs were placed on a Styrofoam raft and floated on liquid nitrogen in a sealed cooler for a minimum of 15 minutes. Samples were then placed in a benchtop freeze dry system (7752000, Labconco, Kansas City, MO, USA) overnight. Prior to observing under SEM, samples were sputter coated with gold alloy (6002-8, Ted Pella, Inc, Redding, CA, USA). Leaf surfaces were observed using a Phenom XL benchtop SEM (Nanoscience Instruments, Phoenix, AZ, USA). Trichome density, as well as counts of sessile glandular, bulbous, and cystolithic trichomes were taken at three independent points measuring 2.88 × 10^5^ μm^2^ on each leaf section. To confirm that sessile trichome morphology was not impacted by sample preparation for SEM, light microscopy images were captured with an Axiocam 105 color camera using a Stemi 508 microscope (Zeiss, Jena, Germany).

### Molecular marker assay design and screening

To resolve the allelic status of the *B* and *O* loci, two polymerase chain reaction (PCR) allelic competitive extension (PACE) assays (3CR Bioscience, Harlow, UK) were developed (Table S1). To distinguish CBG-dominant from CBD-dominant individuals at the *B* locus, *CBDAS* was PCR-amplified using Phusion polymerase (New England Biolabs, Ipswich, MA, USA) and previously developed primers [45] from a CBG-dominant ‘Carmagnola’ × ‘USO-31’ F_2_ individual and subsequently sequenced at the Cornell Biotechnology Research Center using the same primers. PACE assays were then developed for the G to A transition at 1465 bp (Table S1). This assay was designated CH-USO31-IV-1. To distinguish cannabinoid-free from cannabinoid-competent individuals at the *O* locus, the previously described ‘USO-31’-derived low-cannabinoid OLS sequence [41] was aligned to CBDRx LOC115699293, and the C to G transversion at 500 bp with respect to LOC115699293 was developed into a PACE assay (Table S1). This assay was designated CH-OLS-V-1. PACE assays were conducted using the protocol described by Toth et al. [74].

### Detached leaf herbivory bioassay

In order to eliminate confounding variables from the field evaluation, 12 of the clonal genotypes from the ‘Carmagnola’ × ‘USO-31’ F_2_ population were selected for a detached leaf bioassay. Genotypes were selected using HPLC and molecular marker data such that six of the genotypes were cannabinoid-free and six were CBD-dominant. Twelve two-node cuttings were rooted for each genotype using the above protocol and maintained in the greenhouse for 10 weeks under the same conditions as the clonal stock plants. At 10 weeks, the plants were separated into three cohorts for temporal replication, such that there were four individuals of each genotype in each cohort. For each cohort, the middle leaflet from the most recently fully expanded leaf was excised and placed in a 100 × 15 mm polystyrene Petri dish with wet filter paper. Then, one neonate *T. ni* larvae from a colony maintained by the Wang Lab at Cornell University was transferred to the adaxial surface of each leaf. Petri dishes were sealed with Parafilm (Bemis Company, Inc, Neenah, WI, USA) and monitored for larval survival and position in the Petri dish every 24 hours for 6 days. Larval position was observed and recorded in one of four categories: abaxial leaf surface, adaxial leaf surface, petiole, or off leaf. At 6 days, the living larvae were massed using a microbalance and the leaf area consumed was quantified with the LeafByte app [75]. The cohorts were each started 48 hours apart.

### Artificial diet bioassays

To further isolate the impact of cannabinoids on *T. ni* survival and growth, two artificial diet bioassays were conducted. For the first experiment, purified cannabinoids emulsified in glycerin with sunflower lecithin and medium-chain triglyceride oil (Cirona Labs, Geneva, NY, USA) were incorporated into artificial *T. ni* diet (Southland Products, Lake Village, AR, USA) such that the final concentration of cannabinoids was relative to the dry weight of the homogenized diet. The diet was ~77.5% water by weight. Cannabinoid concentrations in the diet were verified by HPLC using the protocol above with the standard 40–50 mg of lyophilized sample. Measured concentrations were between 0.09% and 0.11% for the 0.1% concentration treatments and between 0.95% and 1.05% for the 1% concentration treatments. There were five treatment groups: glycerin control, 0.1% CBDA, 1% CBDA, 0.1% CBGA, and 1% CBGA. There were 24 diet cups per treatment group separated into three temporal replicates, such that there were eight cups of each treatment in each replicate. Seven-day-old *T. ni* larvae that had been reared on unamended artificial diet were massed, and then one larva was placed in each cup of diet. After 7 days, survival was rated and all of the larvae were massed.

For the second experiment, dilutions of the emulsified cannabinoids were prepared such that the concentration reported reflected the concentration of cannabinoids in the emulsion. The emulsions were then painted onto the surface of artificial diet prepared according to the manufacturer’s instructions (84.5% water by weight) in Petri dishes. There were nine treatment groups: glycerin control, 0.001% CBDA, 0.01% CBDA, 0.1% CBDA, 1% CBDA, 0.001% CBGA, 0.01% CBGA, 0.1% CBGA, and 1% CBGA. Nine Petri dishes were prepared for each treatment, and they were split into three temporal replications such that three Petri dishes of each treatment were included in each replicate. Five neonate *T. ni* were transferred onto each of the Petri dishes, and then they were sealed with Parafilm. Larval survival was rated at 3 and 7 days, and larvae living after 7 days were massed.

### Statistical analyses

All statistical analyses were conducted in R version 4.1.3 [76]. Mixed-effect models were fit using lmer from the R package lmerTest [77], unless otherwise stated. The original data used for all analyses can be found in Supplementary Datasets S1–S4.

For the field experiment, a mixed-effects model with cannabinoid chemotype and site as fixed effects, and propagation method as a random effect, was fit to predict AUHPC. Mixed-effects model F-tests were used to determine whether cannabinoid concentration, site, or an interaction between the two influenced herbivore damage in the field. AUHPC was log-transformed to improve the normality of the model residuals. When the main effects were determined to be significant (α = 0.05), a post-hoc multiple comparison using emmeans [78] with Tukey’s distribution for *P*-value adjustment was used to test pairwise differences between chemotype-site combinations. A second mixed-effects model with total cannabinoid concentration and site as fixed effects and propagation method as a random effect was fit to predict AUHPC. Mixed-effects model F-tests were used to determine whether cannabinoid concentration, site, or an interaction between the two influenced herbivore damage in the field. Both the total cannabinoid concentration and the AUHPC were log-transformed to improve the normality of the model residuals. For both models, a Satterthwaite approximation was used to estimate the effective degrees of freedom.

For the detached-leaf bioassay, mixed-effects models with cannabinoid chemotype as a fixed effect and rep as a random effect were fit to predict leaf area consumed and final larval mass for the surviving individuals. Mixed-effects model F-tests were used to determine whether cannabinoid chemotype had a significant effect on leaf area consumed or final larval mass. Additionally, mixed-effect logistic regression models with cannabinoid chemotype as a fixed effect and rep as a random effect were fit using glmer in the R package lme4 [79] to predict mortality and proportion of observations on the abaxial leaf surface. Wald tests were used to determine whether cannabinoid chemotype had a significant effect mortality or proportion of observations on the abaxial leaf surface. For the linear regression models, a Satterthwaite approximation was used to estimate the effective degrees of freedom. For the logistic regression models, a Laplace approximation was used to fit the model.

For the artificial diet bioassays, two one-way ANOVA tests were used to determine if there was an effect of treatment group, replicate, or an interaction between treatment group and replicate on survival and log of final to initial mass ratio for the artificial diet surface and incorporation bioassays, respectively. When the effect of treatment was deemed significant, a post-hoc multiple comparison using emmeans [78] with Tukey’s distribution for *P-*value adjustment was used to test pairwise differences between treatments.

For the trichome count data, two-way ANOVA tests were used to determine if there was an effect of chemotype, leaf surface, or an interaction between chemotype and leaf surface on trichome density for each of the three types of trichomes. When there was a significant interaction between leaf surface and chemotype, a post-hoc multiple comparison using emmeans [78] with Tukey’s distribution for *P-*value adjustment was used to test pairwise differences between treatments.

## Acknowledgements

We are grateful to the research teams from the Smart, Moore, Wang, and Rose labs for their excellent technical assistance on this project, especially Alex Wares, McKenzie Schessl, Natalie McFadden, Joel DeVries, Emily McFadden, Ryan Crawford, Jesse Chavez, Jason Schiller, Wendy Kain, and the farm crews at Cornell AgriTech and the Cornell Agricultural Experiment Station campus area farms. We are also grateful to Dr Kyle Wickings and Dr María Alejandra Gandolfo-Nixon for the use of their lab equipment, Patrick Woods for providing the OLS/TKS gene sequence for the molecular marker design, and to Dr Heather Grab and Dr Brian Nault for advice on the experimental design. This project was funded by grant AC477 from the New York State Department of Agriculture and Markets through Empire State Development.

## Author contributions

G.M.S.: Conceptualization, methodology, formal analysis, investigation, visualization, writing – original draft. S.I.S., J.A.T.: Methodology, investigation. M.A.Q., J.L.C.: Investigation. J.K.M., J.N.J., P.W.: Resources. G.P.: Methodology. J.L.H., V.M.M., J.K.C.R., L.B.S.: Supervision, funding acquisition. All authors contributed to writing—review & editing.

## Data availability

The datasets generated during and analyzed during the current study are available in the supplementary materials.

## Conflict of interest statement

None declared.

## Supplementary data

Supplementary data is available at *Horticulture Research* online.

## Supplementary Material

Web_Material_uhad207
