# Supplementary material for: Cannabinoids function in defense against chewing herbivores in Cannabis sativa L
Source: Hortic Res. 2023 Oct 13;10(11):uhad207. doi: 10.1093/hr/uhad207 (PMC10681003; doi:10.1093/hr/uhad207)
Supplement: Web_Material_uhad207 [file web_material_uhad207.zip › Stack_et_al_Cannabinoids_HortRes_Supporting_Information-2023_09_18.pdf]

George M. Stack[1], Stephen I. Snyder[2], Jacob A. Toth[1], Michael A. Quade[1], Jamie L. Crawford[3], John K. McKay[4], John Nicholas Jackowetz[5], Ping Wang[6], Glenn Philippe[2], Julie L. Hansen[3], Virginia M. Moore[3], Jocelyn K. C. Rose[2], Lawrence B. Smart[1]

[1] Horticulture Section, School of Integrative Plant Science, Cornell University, Cornell AgriTech, Geneva, NY, 14456

[2] Plant Biology Section, School of Integrative Plant Science, Cornell University, Ithaca, NY, 14850

[3] Plant Breeding Section, School of Integrative Plant Science, Cornell University, Ithaca, NY, 14850

[4] Department of Agricultural Biology, Colorado State University, Fort Collins, CO, 80523

[5] Cirona Labs, Geneva, NY, 14456

[6] Department of Entomology, Cornell University, Cornell AgriTech, Geneva, NY, 14456

Cannabinoids function in defense against chewing herbivores in *Cannabis sativa* L.

SUPPLEMENTARY FIGURE & TABLE

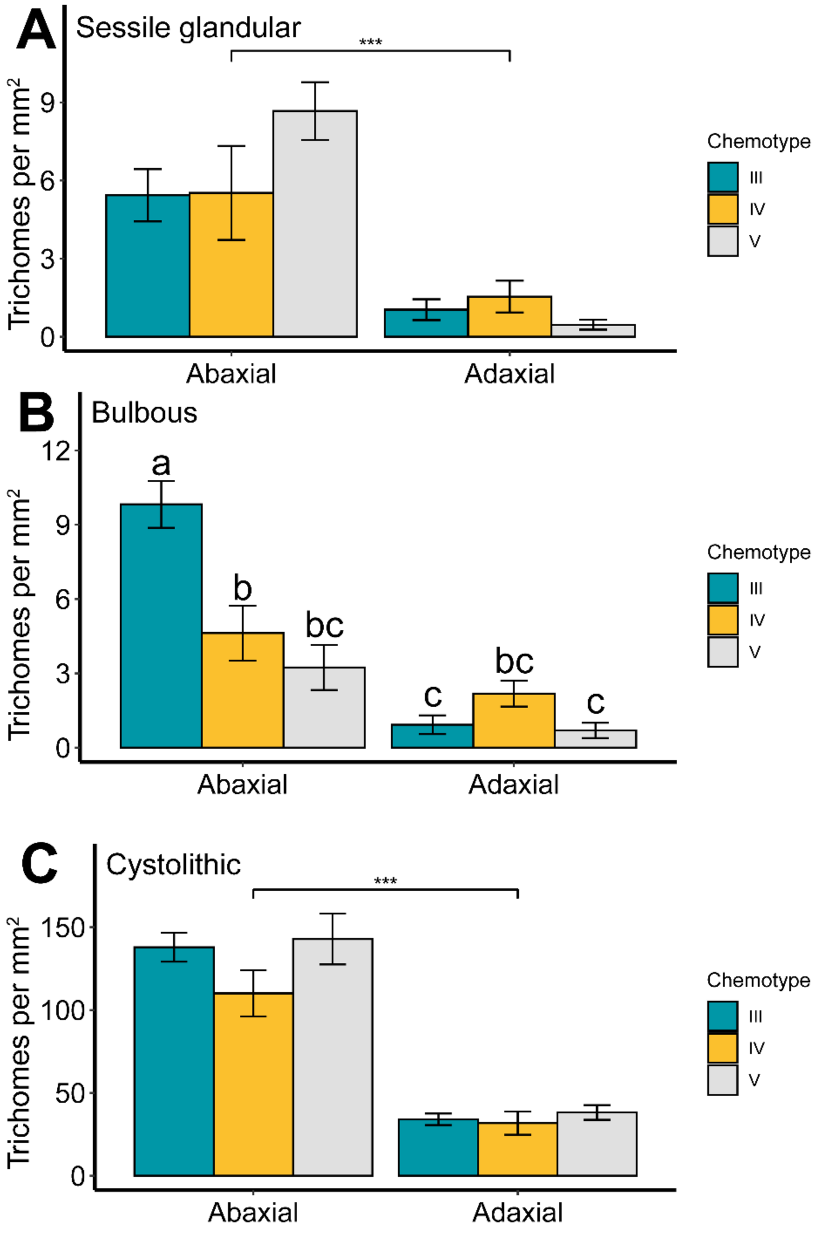

Figure S1. Mean trichome count data on abaxial and adaxial leaf surfaces from representative plants of three cannabinoid chemotypes in a segregating F_2 population of *C. sativa*. Panels depict (A) Sessile glandular trichomes, (B) bulbous trichomes, and (C) cystolithic trichomes. Bars indicate means and error bars indicate standard error. Brackets indicate significant differences (***, p<0.001) between abaxial and adaxial leaf surfaces and letters indicate pairwise differences between chemotype-surface combinations from a Tukey's HSD analysis.

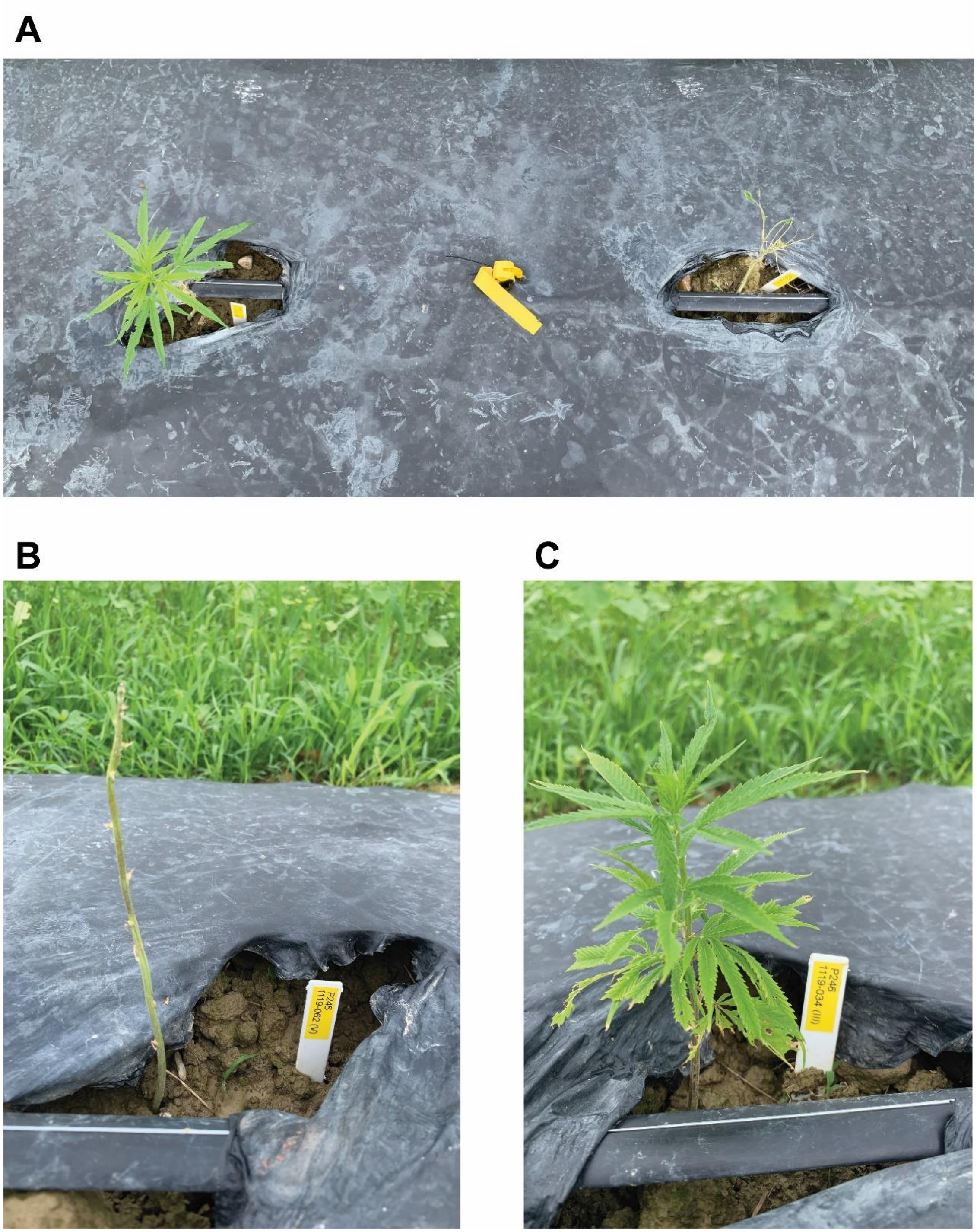

Figure S2. Images of CBD-dominant and cannabinoid-free plants in the Ithaca field site on July 21, 2021. (A) CBD-dominant individual adjacent to a cannabinoid-free individual. (B) Fully defoliated cutting of cannabinoid-free genotype GVA-H-20-1119-062. (C) Cutting of CBD-dominant genotype GVA-H-20-1119-034.

Table S1. Primer sequences for PACE assays used to report alleles at the *B* and *O* loci.

	Sequence (5' to 3')	Description
CH-USO31-IV-1-FAM	GAAGGTGACCAAGTTCATGCTCTCA ATTATAGAGACCTTGATATAG	WT *CBDAS*
CH-USO31-IV-1-HEX	GAAGGTCGGAGTCAACGGATTCTCA ATTATAGAGACCTTGATATAA	'USO-31' *CBDAS*
CH-USO31-IV-1-Common	CCAGGGTTTTCACTTTTACTAGC	Common
CH-OLS-V-1-FAM	GAAGGTGACCAAGTTCATGCTCGCT AAGCTTCTCGGACTC	WT *OLS*
CH-OLS-V-1-HEX	GAAGGTCGGAGTCAACGGATTCGCT AAGCTTCTCGGACTG	'USO-31' *OLS*
CH-OLS-V-1-Common	GGCAATGCGTAGAACGGTTC	Common
